# A Cost-Effective and Easy to Assemble 3D Human Microchannel Blood–Brain Barrier Model and Its Application in Tumor Cell Adhesion Under Flow

**DOI:** 10.3390/cells14060456

**Published:** 2025-03-19

**Authors:** Yunfei Li, Bingmei M. Fu

**Affiliations:** Department of Biomedical Engineering, The City College of the City University of New York, New York, NY 10031, USA; yli012@citymail.cuny.edu

**Keywords:** engineered microvessel, microfluidic device, solute permeability, sodium fluorescein, dextran-70k, glycocalyx, MDA-MB-231, tumor cell adhesion, heparan sulfate

## Abstract

By utilizing polydimethylsiloxane (PDMS), collagen hydrogel, and a cell line for human cerebral microvascular endothelial cells, we produced a 3D microchannel blood–brain barrier (BBB) model under physiological flow. This 3D BBB has a circular-shaped cross-section and a diameter of ~100 μm, which can properly mimic the cerebral microvessel responsible for material exchange between the circulating blood and brain tissue. The permeability of the 3D microchannel BBB to a small molecule (sodium fluorescein with a molecular weight of 376) and that to a large molecule (Dex-70k) are the same as those of rat cerebral microvessels. This 3D BBB model can replicate the effects of a plasma protein, orosomucoid, a cytokine, vascular endothelial growth factor (VEGF), and an enzyme, heparinase III, on either rat cerebral or mesenteric microvessesels in terms of permeability and the modulation of glycocalyx (heparan sulfate). It can also replicate the adhesion of a breast cancer cell, MDA-MB-231, in rat mesenteric microvessels under no treatment or treatments with VEGF, orosomucoid, and heparinase III. Because of difficulties in accessing human cerebral microvessels, this inexpensive and easy to assemble 3D human BBB model can be applied to investigate BBB-modulating mechanisms in health and in disease and to develop therapeutic interventions targeting tumor metastasis to the brain.

## 1. Introduction

The blood–brain barrier (BBB) is a highly selective interface that separates the central nervous system from the peripheral circulation and plays a critical role in maintaining brain homeostasis [1]. Proper in vivo and in vitro BBB models are necessary tools for advancing our understanding of brain physiology, investigating disease mechanisms, and developing efficient drug delivery strategies [2,3,4]. Due to the differences in cerebrovascular structures and compositions between humans and commonly used rodents, animal models often fail to fully replicate human diseases [5,6]. Therefore, in vitro models utilizing human cells serve as a crucial bridge between human and animal studies. They can offer valuable insights into disease mechanisms and aid in the development of novel strategies for drug and gene delivery to the brain [7,8,9,10,11,12,13,14,15].

A 2D BBB model produced on a Transwell filter has been the most widely utilized cell-based in vitro BBB model over the last several decades [2,3,4]. Although the Transwell filter can produce a BBB with comparable permeability to in vivo data, the produced BBB is flat and without the influence of the blood flow. The flow not only more efficiently brings fresh nutrients and carries away the cell-generated waste, it also induces shear stresses, which are important in endothelial responses to various stimulations via mechano-sensors and transcription factors [16,17] and in circulating cell adhesion and transmigration [18,19,20]. Studies by Cucullo et al. [21] found that laminar flow-induced shear stress promotes the formation of a tight and highly selective BBB produced by HBMECs (human brain microvascular endothelial cells) and increases the RNA level of multidrug resistance transporters and ion channels at the BBB. Though the flow can be generated in some 2D BBB models as described in [22,23,24,25,26], they are not able to fully reproduce the 3D BBB anatomical structure, such as the circular-shaped cross-section.

Recent advancements in microfluidics have led to the development of BBB-on-a-chip platforms, which aim to mimic the structural and functional properties of the BBB more closely to cerebral microvessels. Many microfluidic models in use are polydimethylsiloxane (PDMS)-based systems because they confer several advantages: they are biocompatible, inexpensive, easy to manipulate, transparent, and gas-permeable. Devices made from PDMS can be prepared using a photolithography mask, and a replication step allows for the mass-production of the desired structures. They can also be prepared by using 3D-printed molds. However, the main drawback of PDMS, in particular for biomedical applications, is its hydrophobic properties, which may induce the undesired adsorption of organic molecules [27]. In addition, the untreated PDMS surface has a poor affinity for live cells and uncrosslinked free PDMS monomers can leach out into the culture medium and affect cell growth [28]. Due to the nature of the photolithography fabrication process, PDMS-based 3D BBB models usually have microchannels with a rectangular cross-section [29,30]. This geometry results in different flow and shear stress profiles to those in a real, circular-shaped blood microvessel.

In the last several years, many 3D BBB models utilizing PDMS and collagen hydrogel have been produced. Tube-like human BBB models have been produced either from the hollow spaces in 3D hydrogel produced using the needle pull-out method [7,31,32] or within hollow channels in microfluidic systems [33,34,35]. However, the tube-like structures produce vessels that have a diameter of 600–800 μm [36], which is much larger than that of the capillaries and post-capillary venules whose walls are defined as the BBB. Hajal et al. [37] and Winkelman et al. [38] recently developed an in vitro model of the human BBB, self-assembled within microfluidic devices from HBMECs, human brain pericytes, and astrocytes. Zhao et al. [39] also engineered a human BBB at the capillary scale using a double-templating technique. Their approaches could produce microvessels with a diameter of 10–40 μm. However, their techniques are quite complicated and there is a need to fabricate the devices in a clean room. Linville et al. [7] produced a tube-like BBB with a diameter of ~150 μm made from PDMS and hydrogel using the needle pull-out method. Although their manufacturing procedure is less complicated than the self-assembly method, it still needs relatively high skill and sophisticated apparatus. In addition, the high cost of these 3D BBB systems limits their widespread use, especially in labs with budget constraints [21]. Therefore, the main aim of this study was to produce a simple, easy to assemble, and cost-effective 3D microchannel BBB with a circular cross-section and a diameter of ~100 μm, mimicking the flow and shear stress profiles in real microvessels, particularly in the post-capillary venules in the brain. This inexpensive 3D BBB can be constructed in a general wet lab without using a clean room or other microfabrication techniques.

Collagen type I is a popular hydrogel used in many models of the microvasculature due to its biocompatibility and physiological resemblance to the natural extracellular matrix (ECM). In this study, a 3D human BBB-on-chip in PDMS–hydrogel was produced. The hydrogel made of 4–5 mg/mL collagen I simulated brain tissue, with comparable mechanical and transport properties [7,40,41,42]. In the current study, the collagen gel-based 3D microtube structure was constructed by using a microneedle with a diameter of ~100 μm. After the 3D microchannel BBB was produced using hCMECs (human cerebral microvascular endothelial cells) under a physiological flow, its permeability to small and large solutes was quantified, as well as its surface glycocalyx, one of the crucial components in regulating the BBB’s function. As an application of the produced 3D BBB, the effects of glycocalyx (heparan sulfate, HS)-modulating agents on the modulation of HS and the permeability of the 3D microchannel BBB were evaluated. To test the HS-modulating effects on tumor cell metastasis in the microcirculation, the adhesion of a malignant breast cancer cell, MDA-MB-231 (or MB231), in the 3D BBB under flow was quantified with no treatment and after treatments with HS-modulating agents.

## 2. Materials and Methods

### 2.1. Cell Culture

Human cerebral microvascular endothelial cells (hCMECs/D3 or hCMECs) from Millipore Sigma (Burlington, MA, USA) (passage 8 to 19 after purchase) were cultured in EBM^TM^-2 Basal Medium (Lonza, Basel, Switzerland), also using an EGM^TM^-2 MV Microvascular Endothelial Cell Growth Medium SingleQuots^TM^ kit (Lonza) [43,44]. Human breast carcinoma cells, MDA-MB-231 (or MB231), from ATCC (Manassas, VA, USA) (passage 11 to 16 after purchase) were cultured in Dulbecco’s Modified Eagle’s Medium/Nutrient Mixture F-12 Ham (DMEM/F-12) (Sigma-Aldrich, St. Louis, MO, USA), supplemented with 2 mM L-glutamine (Sigma-Aldrich, St. Louis, MO, USA), 100 U/mL penicillin (Sigma-Aldrich, St. Louis, MO, USA) and 1 mg/mL streptomycin (Sigma-Aldrich, St. Louis, MO, USA), and 10% fetal bovine serum (FBS, Atlanta Biologicals, Flowery Branch, GA, USA) [44,45]. All cells were cultured in the incubator with 5% CO_2_ at 37 °C.

### 2.2. The Production of a 3D PDMS–Hydrogel Microchannel

**Figure 1:**
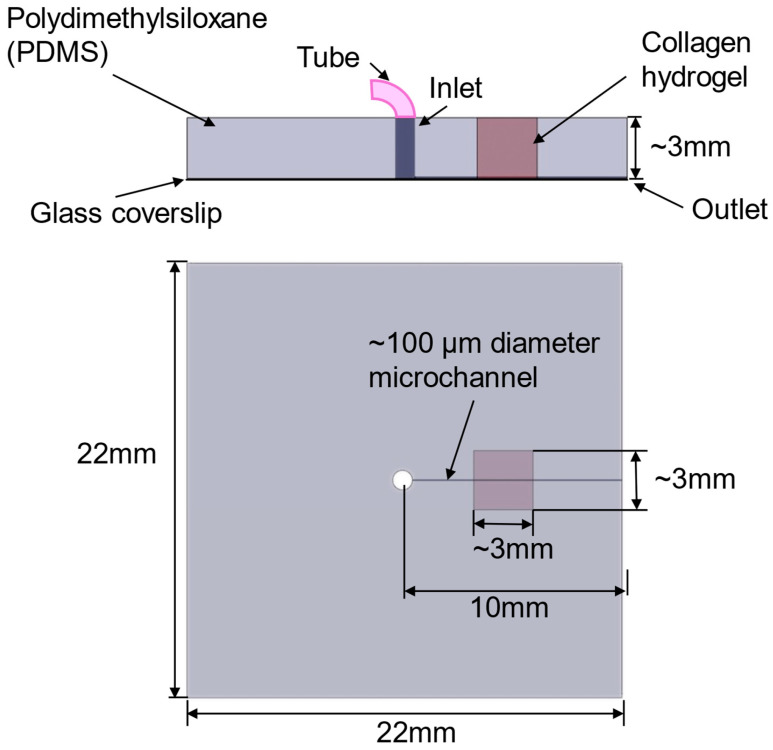
Schematic of the PDMS–collagen hydrogel microchannel device. PDMS solidification on a glass coverslip to form a 22 mm × 22 mm × ~3 mm base. A microchannel with a diameter of ~100 μm was formed by pulling out a microneedle from the collagen hydrogel. The inlet was formed with an 18-gauge tubing adapter and connected to PE-50 tubing for perfusion, which was driven by a syringe pump. Step-by-step instructions for producing the PDMS–collagen gel microchannel device was given in Supplement S1.

A polydimethylsiloxane (PDMS; Sylgard, Dow Corning, Midland, MI, USA) pre-polymer was mixed with a curing agent (10:1 *w*/*w* ratio of PDMS to the curing agent). The bubbles generated during the mixing were removed in a vacuum chamber by setting the mixture in the chamber for at least 30 min. Then, the liquid PDMS was poured on top of a clean glass coverslip (22 × 22 mm, Dow Corning) with a 120 μm diameter acupuncture needle (SEIRIN, Thermo Fisher Scientific, Waltham, MA, USA) with a length of 10 mm in a hexagon container. The coverslip was thoroughly cleaned using acetone or ethanol followed by deionized water and dried with a high-speed airflow. Before being placed on the coverslip, the needle was coated with the 1% BSA/PBS mixture overnight at 4 °C. The PDMS mixture was cured for 35 min at 100 °C in an oven or overnight in a 37 °C incubator. After solidification, the needle was pulled out to produce a microchannel in the PDMS base. An 18G tubing adapter was used to drill a hole perpendicularly in the PDMS base to form an inlet (Figure 1). A segment of PDMS (~3 × 3 mm) in the middle of the microchannel was removed and the needle was reinserted. Next, neutralized collagen type I from a rat tail (Advanced Biomatrix, Thermo Fisher Scientific, Waltham, MA, USA) was created at a concentration of ~5 mg/mL [7,46]. A total of 1 mM genipin (Sigma-Aldrich St. Louis, MO, USA) was also added for the crosslinking to stabilize the collagen hydrogel [47]. The collagen mixture was then added to the cut segment of the PDMS. After incubation overnight at 37 °C, the needle was pulled out to create a microchannel in the collagen part of the device. The inlet was connected to PE-50 tubing (BD, Thermo Fisher Scientific, Waltham, MA, USA) for perfusion, which was driven by a syringe pump (NE-1800, New Era Pump system, Farmingdale, NY, USA). Then, the PBS solution was perfused into the microchannel for at least 1 h to remove the residual genipin [48], followed by perfusion with a cell culture medium overnight. Figure 1 shows the schematic of the produced PDMS–collagen hydrogel microchannel device. The distance from the microchannel to the coverslip was estimated to be ~5 μm. Kim et al. [46] found that collagen I at a concentration of 4–5 mg/mL has an elastic modulus similar to that of a mouse hippocampus. Grifno et al. [49] found that 7 mg/mL collagen crosslinked with genipin resulted in a Young’s modulus of ~3 kPa, which is comparable to that of mouse brain tissue. Chrobak et al. [50] also reported that the gel formed by collagen with a concentration of 5–7 mg/mL could prevent the infiltration of cells into the gel.

**Figure 2:**
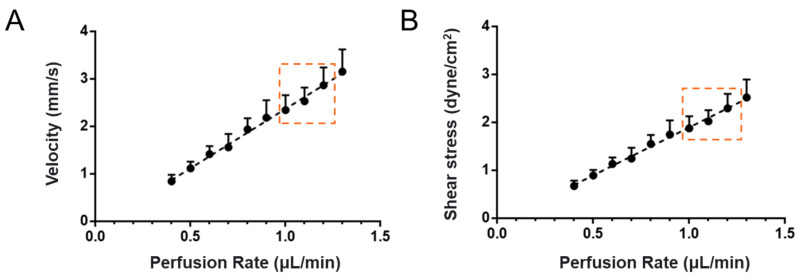
Calibration plots for the perfusion rate vs. the velocity (**A**) and the perfusion rate vs. the wall shear stress (**B**) in a circular microchannel with a diameter of ~100 μm. The range of perfusion rates for generating appropriate physiological wall shear stresses in post-capillary venules is indicated by the region enclosed with an orange dashed line.

The flow rate was set up to generate 1–3 dyne/cm^2^ wall shear stress (WSS) for a microchannel with a diameter of about 100 μm, which is the same as the physiological conditions in the post-capillary venule of cerebral microcirculation [7,51,52,53]. Figure 2 presents the relation between the perfusion rate and the flow velocity and that between the perfusion rate and the WSS. The equation to calculate the WSS is given by τ = (4 μV)/D, where τ is the wall shear stress, μ is the dynamic viscosity of the perfusate, which was estimated as 2 mPaS for 1 million cells/mL in 1% BSA–Ringer solution or the cell culture medium [45], V is the flow velocity in the channel, and D is the microchannel diameter. The calibration relation shown in Figure 2A was performed by tracing a cell tracker red-labeled MB231 cell with a Nikon imaging system. The flow velocity was estimated as the centerline velocity of an MB231 cell [54]. The perfusion rate of 1–1.3 μL/min in our system could generate the same WSS as in the post-capillary venule of the cerebral microcirculation (Figure 2B). Guo et al. [55] found that MB231 cells prefer to adhere to the wall of post-capillary venules after overcoming the mechanical trappings of capillaries with a diameter of less than 10 μm.

### 2.3. The Generation of a 3D Microchannel BBB Under Flow

The suspension of hCMECs at four to five million cells/mL was made in an EGM^TM^-2 MV culture medium (Lonza, Basel, Switzerland), along with 8% dextran-70k, which could increase the viscosity of the loading medium and promote vascular stability [56,57]. Then, the cell suspension was slowly perfused into the microchannel via the inlet using a pipettor with a 10 µL pipette. After checking with a microscope to make sure that the cells were attached in the microchannel, the device was incubated and flipped over every 15 min to ensure cells were seeded evenly on the microchannel surface. After 1 h of incubation, the suspension medium was replaced with a fresh EGM^TM^-2 MV medium and the microchannel was linked to a PE50 tubing system and perfused at a rate of 1.0–1.3 μL/min using a syringe pump, starting at 6 h after seeding. The 3D microchannel BBB was formed in 4–5 days under this physiological flow (Figure 2B).

Step-by-step instructions for producing the 3D microchannel BBB are presented in Supplement S1.

### 2.4. Quantification of Heparan Sulfate (HS) in 3D Microchannel BBB

**Figure 3:**
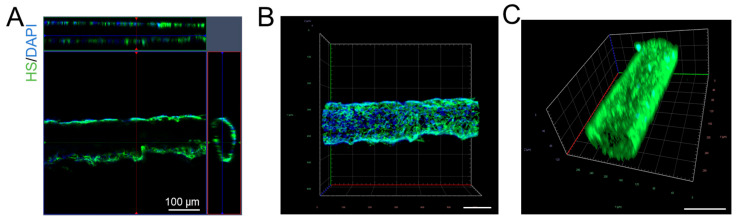
Confocal images showing the 3D microchannel BBB labeled with anti-HS (heparan sulfate) and DAPI. (**A**) Multiview orthographic projections, (**B**) top-perspective view, and (**C**) 3D view of the 3D BBB labeled with anti-HS. The scale bar in (**B**,**C**) is 100 μm.

To verify that glycocalyx (HS) was successfully produced in the 3D microchannel BBB and to quantify the effects of orosomucoid, VEGF, and heparinase III on the modulation of HS in the microchannel BBB, the microchannel BBB was immuno-stained with FITC–anti-HS as described in [45]. Briefly, the microchannel BBB was first rinsed with 10 mg/mL bovine serum albumin (BSA, Sigma-Aldrich, St. Louis, MO, USA) in PBS (1% BSA/PBS) and fixed with 2% paraformaldehyde and 0.1% glutaraldehyde for 20 min. Then, 0.1% NaBH_4_ (Sigma-Aldrich, St. Louis, MO, USA) was used to treat the BBB for 7 min. After being rinsed with 1% BSA/PBS, the BBB was blocked by 2% normal goat serum for 30 min at room temperature (RT). Then, the HS on the microchannel BBB was labeled with an FITC-conjugated antibody (1:100) at 4 °C for ~2.5 h [45]. The 2.5 h period was long enough to allow FITC–anti-HS to infiltrate the entire depth of the glycocalyx [58]. After being rinsed with 1% BSA/PBS to remove the free dye, the microchannel BBB was scanned using a Zeiss LSM 800 confocal laser scanning microscope (Carl Zeiss Microscopy, LLC, White Plains, NY, USA) with a 20×/NA0.8 objective lens. Two fields (each field was 465 µm × 465 µm) (2048 × 2048) from each microchannel were captured as a z-stack of 20–30 images with a z-step of 5.28 μm. Image projection and intensity quantification for HS were performed using Zeiss ZEN desk and NIH ImageJ (version 1.53t). Figure 3 shows confocal images of the 3D microchannel BBB labeled with FITC–anti-HS and DAPI.

### 2.5. Modulation of HS of 3D BBB and MB231 by Various Agents

By applying heparinase III at a variety of concentrations for 2 h, Zeng et al. [59] degraded HS on the rat fat pad endothelial cell monolayer. Cai et al. [45] disrupted HS on the luminal surface of a microvessel in vivo by perfusing 1% BSA–Ringer solution with 50 mU/mL heparinase III into a rat mesenteric post-capillary venule for 1 h. Accordingly, we applied 50 mU/mL heparinase III (Sigma-Aldrich) for 2 h to manipulate the HS on the 3D BBB and MB231 cells before investigating MB231 adhesion to the BBB. Xia et al. [43] applied 1 nM VEGF for 2 h to a 2D BBB and MB231 cells to demonstrate a differential effect on their respective HS quantities. Shen et al. [60] pretreated a microvessel with 1 nM VEGF for 1 h to investigate tumor cell adhesion to the wall of a microvessel on a rat mesentery. They showed that VEGF increased tumor cell adhesion. Correspondingly, we pretreated the 3D BBB and MB231 cells with 1 nM VEGF (recombinant human VEGF_165_, Peprotech, Rocky Hill, NJ, USA) for 2 h. By perfusing 0.1 mg/mL orosomucoid in 1% BSA–Ringer solution into a rat mesenteric post-capillary venule for 30 min, Cai et al. [45] enhanced HS in the microvessel to 1.4 folds that of the control. Therefore, we pretreated the 3D BBB and MB231 cells for 1 h with 0.1 mg/mL orosomucoid (G3643, α1-acid glycoprotein from bovine plasma, Sigma-Aldrich). The method for modulating the quantity of HS on the 3D BBB was the same as for the 2D BBB which was introduced in [44]. The only difference was that the treatment was under physiological flow in the 3D BBB, while it was static in the 2D BBB.

### 2.6. Quantification of 3D Microchannel BBB Permeability

**Figure 4:**
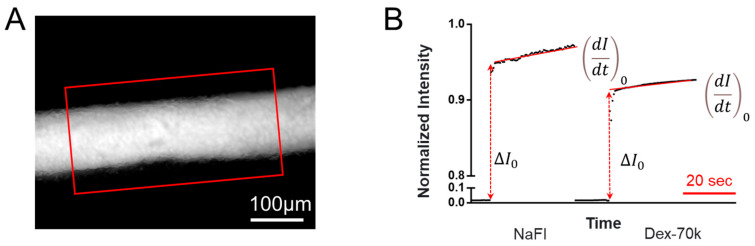
Schematic for determining the permeability of the 3D microchannel BBB to fluorescently labeled solutes. (**A**) Fluorescent microscopic image showing a 3D microchannel BBB filled with fluorescently labeled solutes. (**B**) Total fluorescence intensity in the ROI (the region enclosed by the red frame in (**A**)) as a function of the perfusion time. Fluorescence intensity in the ROI is proportional to the total amount of solutes in the ROI. The slope of the intensity vs. time curve during the initial perfusion (indicated by a red line) is (dI/dt)_0_, which is used to calculate the solute permeability of the microchannel BBB: P = 1/ΔI_0_ × (dI/dt)_0_ × r/2, where ΔI_0_ (red dotted line with arrowheads) is the step increase in the intensity in the ROI when the fluorescently labeled solutes just fill up the lumen of the microchannel BBB, and r is the radius of the vessel. The time scale bar in the figure is 20 s. The smaller the solute (NaFl), the larger the slope. Detailed instructions for how to determine the solute permeability of a microchannel wall are given in Supplement S2.

**Figure A1:**
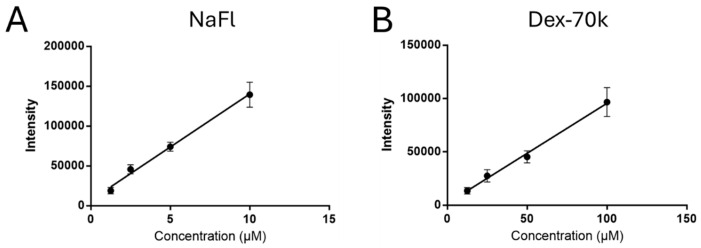
Calibration for the concentration vs. intensity curves for NaFl (**A**) and FITC-Dex-70k (**B**) using the imaging system to determine the solute permeability of the 3D microchannel BBB.

The method for quantifying the solute permeability of the 3D microchannel BBB was the same as that described in refs. [61,62,63]. Briefly, 5 μM sodium fluorescein (NaFl, MW of 376, Sigma-Aldrich) or 50 μM FITC–dextran-70k (Dex-70k, MW of 70 kD, Sigma-Aldrich) in 1% BSA–Ringer solution was perfused into the microchannel BBB at a flow rate of ~1.5 μL/min. The calibration of the NaFl and FITC-Dex-70k concentration vs. intensity curves using our imaging system for the permeability measurement is shown in Figure A1. The quantities of 5 μM for NaFl and 50 μM for FITC-Dex-70k used in the permeability measurement were in the linear range of the calibration curve. Simultaneously, a Nikon TE2000-S imaging system with a 20×/NA0.75 objective lens was used to capture an image every 0.25–0.5 s for 5–60 s. NIH ImageJ (version 1.53t) was used for measuring the intensity profiles of the ROI (region enclosed by the red line) shown in Figure 4A. The permeability (P) to NaFI or Dex-70k was calculated by using the following equation:

$$P=\frac{1}{{∆I}_{0}}(\frac{dI}{dt}{)}_{0}\frac{r}{2}$$

where ${∆I}_{0}$ is the fluorescent intensity in the ROI when the dye just fills up the lumen of the microchannel BBB, ${\left(\frac{dI}{dt}\right)}_{0}$ is the slope of the intensity vs. time curve during the initial perfusion, and *r* is the radius of the microchannel BBB (Figure 4B). To determine the effect of HS modulation on the permeability of the microchannel BBB, VEGF, orosomucoid and heparinase III were used to treat the microchannel BBB. To determine the effect of orosomucoid or heparinase III, after measuring the control permeability (P), 0.1 mg/mL orosomucoid or 50 mU/mL heparinase III in 1% BSA–Ringer solution was injected into the microchannel BBB for 1 h or 2 h, respectively; then, the P was measured in the same microchannel BBB after the treatment. Since 1 nM VEGF has an acute effect on the permeability of the BBB in vivo in rat cerebral microvessels [64], to test for an acute effect of VEGF on the in vitro 3D BBB, after the baseline P measurement, NaFl or FITC-Dex-70k in 1 nM VEGF in 1% BSA–Ringer solution was continuously perfused into the microchannel BBB, and an image was collected every 0.25 s for 8 min. The permeability of the microchannel BBB was calculated every 30 s.

### 2.7. The Quantification of MB231 Cell Adhesion to the 3D Microchannel BBB Under Flow

For the tumor cell adhesion experiments, MB231 cells were first fluorescently labeled with 10 μM cell tracker red, EX/EM = 577/602 nm (Invitrogen, Thermo Fisher Scientific), for 30 min and filtered using a 40 µm cell strainer before being perfused into the 3D microchannel BBB [45]. Under no treatment, pretreatment with 50 mU/mL heparinase III applied for 2 h to the microchannel BBB only or to both the BBB and MB231 cells, or pretreatment with 1 nM VEGF applied for 2 h and 0.1 mg/mL orosomucoid applied for 1 h to both the BBB and MB231 cells, as described by Li et al. [44], MB231 cells at a concentration of ~1 million/mL in 1% BSA–Ringer solution were perfused into the microchannel BBB at a 1–1.3 μL/min flow rate for 1 h at 37 °C. The microchannel BBB with adherent MB231 cells was imaged using a Nikon Eclipse TE2000-S microscope (Nikon instrument Inc., Melville, NY, USA) with a 20×/NA0.75 objective lens [45]. Two fields (436 μm × 334 μm for each field) were imaged for each sample with 3 independent experiments analyzed for each case. The number of adherent MB231 cells was counted for each case and presented as the number per 50,000 μm^2^ of the mid-plane area of the microchannel segment.

### 2.8. Statistical Analysis

The data were presented as the mean ± the standard deviation (SD) for all the measurements. A *T*-test or two-way ANOVA was used for comparisons between treatment and no-treatment conditions and among different treatments. The samples were from at least 3 independent experiments. A *p*-value < 0.05 was considered statistically significant for all the experiments.

## 3. Results

### 3.1. Comparison of the Solute Permeability of the 3D Microchannel BBB with That of the 2D BBB and That of Rat Cerebral Microvessels

**Figure 5:**
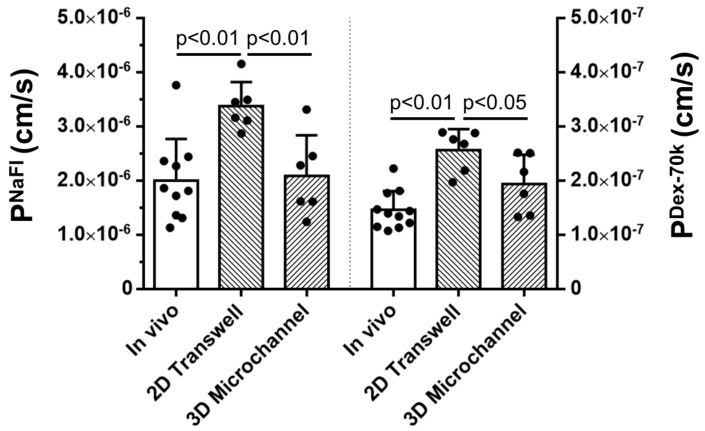
Comparison of the P^NaFl^ and P^Dex-70k^ of the in vitro 2D Transwell BBB, 3D microchannel BBB, and in vivo BBB. The in vivo data are from the measured permeability of rat cerebral microvessels (Shin et al., 2020, ref. [65]). *n* ≥ 6 samples/vessels for each case. Values are the mean ± the SD.

Unlike for the 2D BBB produced on a Transwell filter [44], it was hard to measure the TEER (indicator of the BBB’s permeability to ions or small molecules) for the 3D microchannel BBB; instead, the permeability (P) to a small molecule, sodium fluorescein (NaFl, MW = 376) (P^NaFl^) was quantified. Figure 5 shows that the P^NaFl^ of the microchannel BBB was 2.09 ± 0.75 × 10^−6^ cm/s, which is not significantly different from that of rat cerebral microvessels, 2.00 ± 0.76 × 10^−6^ cm/s [65] (*p* > 0.83), but significantly smaller than that of the 2D BBB, 3.37 ± 0.44× 10^−6^ cm/s (*p* < 0.007). Similarly, the P to a large molecule, Dex-70k (P^Dex-70k^), of the microchannel BBB was 1.94 ± 0.53 × 10^−7^ cm/s, which is not significantly different to that of rat cerebral microvessels, 1.46 ± 0.35 × 10^−7^ cm/s [65] (*p* > 0.08), but significantly smaller than that of the 2D BBB, 2.56 ± 0.38 × 10^−7^ cm/s (*p* < 0.05). These results indicate that the flow plays a crucial role in producing an in vivo-like BBB.

### 3.2. Effects of Heparinase III, VEGF, and Orosomucoid on the HS Modulation in the 3D Microchannel BBB

**Figure 6:**
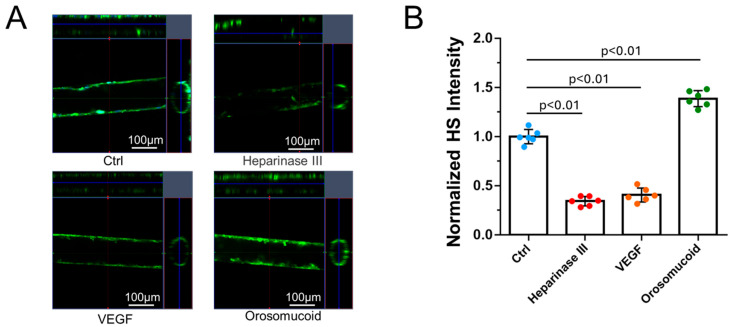
Modulation of the amount of heparan sulfate (HS) in a 3D microchannel BBB by various agents. (**A**) Confocal images showing HS in the BBB under control conditions and after treatment with various agents. (**B**) Normalized HS intensity of the BBB under control (Ctrl) conditions and various treatments. Values are the mean ± the SD. *n* = 3 samples with 2 fields (each field was 465 µm × 465 µm) per sample analyzed for each case.

**Figure A2:**
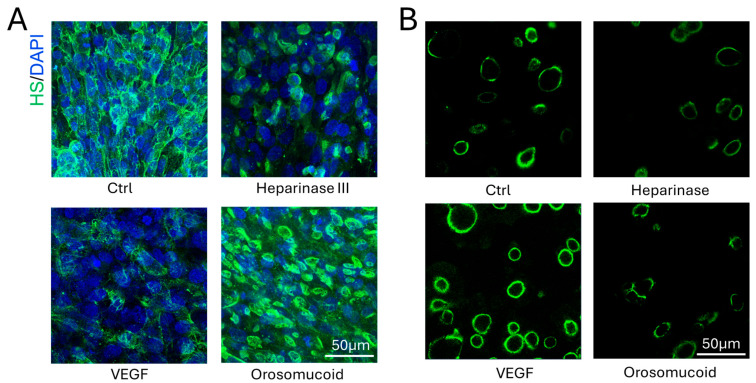
Modulation of heparan sulfate (HS) in a 2D BBB (**A**) and in MB231 cells (**B**) by various agents. From Li et al., 2024 [44].

To investigate the effects of HS-modulating agents on the 3D microchannel BBB produced under flow, in the same way as for the 2D BBB generated on Transwell filters under static conditions, we treated the 3D BBB with 50 mU/mL heparinase III or 1 nM VEGF for 2 h or 0.1 mg/mL orosomucoid for 1 h under flow. Figure 6 shows confocal images (Figure 6A) and the intensity quantification of the HS (Figure 6B) in the microchannel BBB under no treatment (control) and after treatments with these HS-modulating agents. Figure 6A shows the FITC–anti-HS images of the middle plane of the microchannel BBB with side views in certain sections under various conditions. For each condition, the total intensity of the images from the z-stack was determined using NIH ImageJ and normalized by the intensity under control conditions (Figure 6B). Heparinase III and VEGF reduced the amount of HS in the microchannel BBB to 34.3 ± 4% and 40.6 ± 7% of the control, respectively, comparable to but different from their effects on HS in the 2D BBB, which resulted in HS amounts of 63 ± 8% (*p* < 0.001) and 29 ± 6% (*p* < 0.01) of the control [44], respectively (see also Figure A2A). In contrast, orosomucoid increased the amount of HS in the microchannel BBB to 1.4 ± 0.1 folds that of the control, which is the same as the effect on the amount of HS in post-capillary venules in a rat mesentery [45]. However, orosomucoid increased the amount of HS in the 2D BBB produced on the Transwell filter under static conditions to 3.4 ± 0.6 folds that of the control, 2.4 folds that for the 3D BBB. It seems that static conditions favor the HS-enhancing effect of orosomucoid.

### 3.3. Effects of Heparinase III, Orosomucoid, and VEGF on the Solute Permeability of the 3D Microchannel BBB

**Figure 7:**
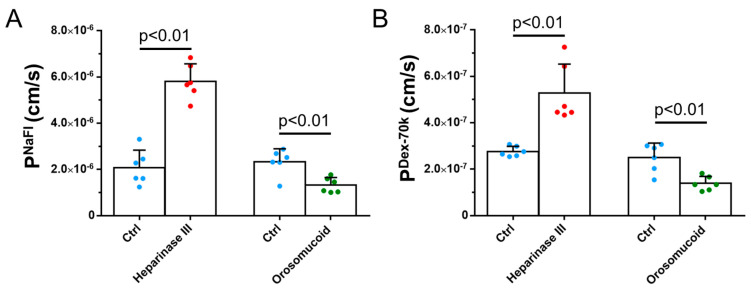
Effects of heparan sulfate (HS) modulation by various agents on the 3D microchannel BBB’s permeability to NaFl, P^NaFl^ (**A**), and dextran-70k, P^Dex-70k^ (**B**). Values are the mean ± the SD. *n* = 6 samples from 3 independent experiments for each case.

To investigate the effect of HS modulation on the barrier function of the 3D BBB, we measured the solute permeability (P) of the 3D BBB to a small molecule, sodium fluorescein (NaFl), P^NaFl^, and to a large molecule, Dex-70k, P^Dex-70k^. Figure 7 shows that, after the reduction in HS caused by heparinase III, the P^NaFl^ increased to 2.8 folds, from 2.09 ± 0.75 × 10^−6^ cm/s to 5.81 ± 0.75 × 10^−6^ cm/s, and the P^Dex-70k^ increased to 1.9 folds, from 2.77 ± 0.22×10^−7^ cm/s to 5.27 ± 0.12 × 10^−7^ cm/s. In contrast, orosomicoid enhanced HS production and decreased the P^NaFl^ to 56.7%, from 2.33 ± 0.56 × 10^−6^ cm/s to 1.32 ± 0.33 × 10^−6^ cm/s, and decreased the P^Dex-70k^ to 55.2%, from 2.51 ± 0.61 × 10^−7^ cm/s to 1.39 ± 0.31 × 10^−7^ cm/s. The effects of heparinase III and orosomucoid on the P^Dex-70k^ of the 3D BBB were comparable to those on the P^Dex-70k^ of the 2D BBB [44].

**Figure 8:**
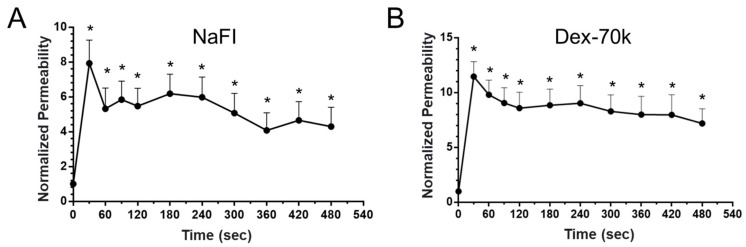
Acute effects of 1 nM VEGF on microchannel BBB permeability. Normalized permeability to NaFl (**A**) and dextran-70k (**B**) by baseline value as function of time. * *p* < 0.05 compared with baseline. Values are mean ± SD. n = 6 samples from 3 independent experiments for each case.

It was found that VEGF has an acute effect on the permeability (P) of rat cerebral microvessels [64]. We tested this acute effect of VEGF on the permeability of the 3D BBB. Figure 8 shows that for both small and large molecules, there was a transient increase in the P within 30 s, peaking at 30 s. The peak P^NaFl^ was 1.7 × 10^−5^ cm/s, a 7.9-fold increase from the baseline P of 2.1 × 10^−6^ cm/s (*p* < 0.001). Unlike in rat cerebral microvessels, the P^NaFl^ did not return to the baseline in 2 min. Instead, it remained at ~6 folds that of the baseline at 2 min and ~5 folds at 8 min (*p* < 0.01), at which time, we ended the measurement. The same pattern applied to P^Dex-70k^, which peaked at 30 s with a value of 2.7× 10^−6^ cm/s, an 11.4-fold increase from its baseline P of 2.4 × 10^−7^ cm/s (*p* < 0.001). It remained at ~9 folds that of the baseline at 2 min and ~7 folds at 8 min (*p* < 0.02).

### 3.4. Effects of HS Modulation on MB231 Adhesion to 3D Microchannel BBB Under Flow

**Figure 9:**
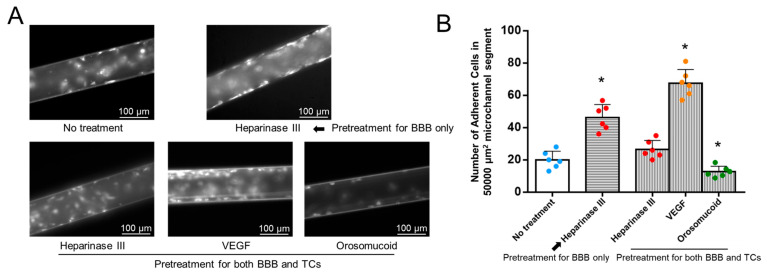
Effects of heparan sulfate (HS) modulation by various agents on MB231 adhesion to 3D microchannel BBB under flow. (**A**) Fluorescent microscopic images of MB231 cells adherent to BBB under control conditions and after various treatments. (**B**) Number of MB231 cells adherent to 3D microchannel BBB under no treatment (control) and after various treatments. * *p* < 0.05, comparison between labeled case and control (no treatment). Values are mean ± SD. *n* = 6 fields (436 µm × 334 µm for each field) from 3 independent experiments analyzed for each case.

Our recent study [44] reported that heparinase III reduced the amount of HS on both MB231 cells and the BBB; however, VEGF and orosomucoid had differential effects on the HS production of MB231 cells and the BBB. While VEGF increased the HS production of MB231 cells, it decreased that of the BBB. In contrast, orosomucoid decreased the HS production of MB231 cells, but it increased that of the BBB. Figure A2 from the study by Li et al. [44] summarizes these effects. Based on their observations, to investigate the effects of HS modulation on MB231 adhesion to the 3D microchannel BBB under flow, we included four types of modulation: (1) only the 3D BBB was pretreated with heparinase III to reduce the HS production of the BBB but keep the HS production of MB231 cells intact; (2) both MB231 cells and the BBB were pretreated with heparinase III to reduce the HS production of both MB231 cells and the BBB; (3) both MB231 cells and the BBB were pretreated with VEGF, which enhances the HS production of MB231 cells but reduces that of the BBB; and (4) both MB231 cells and the BBB were pretreated with orosomucoid, which reduces the HS production of MB231 cells but enhances that of the BBB. Figure 9 shows the effects of HS modulation on MB231 adhesion to the microchannel BBB under flow. Figure 9A demonstrates typical fluorescent microscopic images of MB231 cells adherent to the BBB under various pretreatments. Figure 9B presents the quantification results. Under the no-pretreatment condition, 20 ± 5 MB231 cells were adherent to the microchannel BBB per 50,000 μm^2^ of the mid-plane area of a microchannel segment. For the first modulation performed by pretreating only the BBB with heparinase III, the adherent MB231 cells increased by 2.3 folds compared to with no treatment, indicating that reducing the HS production of the BBB increases MB231 adhesion. In the second modulation performed by pretreating both the BBB and MB231 cells with heparinase III, the number of adherent MB231 cells did not differ from that with no treatment, indicating that reducing HS production in both the BBB and MB231 cells neutralizes the effect on MB231 adhesion. For the third modulation performed by pretreating both the BBB and MB231 cells with VEGF, the adherent MB231 cells increased by 3.4 folds compared to with no treatment, indicating that enhancing the HS production of MB231 cells but reducing that of the BBB favors MB231 adhesion. Finally, for the fourth modulation performed by pretreating both the BBB and MB231 cells with orosomucoid, the MB231 adhesion reduced to 63% of that with no treatment, indicating that reducing the HS production of MB231 but enhancing that of the BBB retards MB231 adhesion.

For MB231 adhesion to the 2D BBB under static conditions, pretreatment applying heparinase III to the BBB only increased the MB231 adhesion by 1.3 folds, and pretreatment applying VEGF to both the BBB and MB231 cells increased the adhesion by 2.3 folds, while pretreatment applying orosomucoid to both the BBB and MB231 cells reduced the adhesion to 68%. Compared with these data obtained under static conditions, the effects of HS modulation on MB231 adhesion appear to have been augmented by the physiological flow.

## 4. Discussion

By utilizing PDMS, collagen hydrogel, and a cell line for human cerebral microvascular endothelial cells (hCMECs), we produced a 3D microchannel BBB under physiological flow. This 3D BBB had a circular-shaped cross-section and a diameter of ~100 μm, which could properly mimic a cerebral microvessel, i.e., a post-capillary venule, responsible for material exchange between the circulating blood and brain tissue (the blood–brain barrier). The permeability of the 3D microchannel BBB to a small molecule (sodium fluorescein with a molecular weight of 376) and that to a large molecule (Dex-70k) were the same as what were measured in vivo for rat cerebral microvessels [64,65]. They were smaller than those of the 2D BBB using the same hCMECs but produced on the Transwell filter under static conditions [44]. Our results indicate that a proper flow is crucial in generating an in vivo-like BBB. The flow or flow-induced shear stress effect was consistent with other studies. Cucullo et al. [21] reported that shear stress increases the TEER and RNA levels of a variety of tight and adherent junctions, resulting in enhanced BBB integrity. Winkelman et al. [38] also found that interstitial flow enhances the barrier function of the 3D microvascular networks generated within a microfluidic device.

Since glycocalyx contributes significantly to the function of the BBB, including modulating the BBB’s permeability to water and solutes and serving as the barrier between circulating cells and the endothelium as well as a mechano-sensor for the blood flow [17,66], we examined the amount of glycocalyx in the 3D microchannel BBB produced under flow. The same as for the 2D BBB, this 3D BBB demonstrated a significant amount of glycocalyx, specifically, heparan sulfate (HS), comparable to what has been observed in rat mesenteric microvessels [45,58]. The modulation of the HS production in the 3D BBB, as well as its permeability, by VEGF, heparinase III, and orosomucoid were also similar to what has been found in either rat mesenteric microvessels [45,60] or rat cerebral microvessels [64]. The transient increase caused by VEGF observed in rat cerebral microvessels shows that exposure to 1 nM VEGF transiently increased the permeability to sodium fluorescein (P^NaFl^) and that to Dex-70k (P^Dex-70k^) to 2.2 and 9.8 times their control values, respectively, within 30 s, and both returned to the control values in 2 min. Unlike the 2D BBB, in which the transient effects of VEGF on its permeability were not able to be measured, the 3D BBB enabled the measurement of the transient response. Although it was observed that the exposure of the 3D BBB to 1 nM VEGF transiently increased the P^NaFl^ and P^Dex-70k^ to 7.9 folds and 11.4 folds that of their control values in 30 s, respectively, the increased P^NaFl^ and P^Dex-70k^ did not return to the control values in 2 min or even in 8 min, after which the measurement was stopped. The increases were maintained at 5 folds and 7 folds of their baselines, respectively. The reason for this observation is unknown but possibly was because the current form of the 3D BBB does not include pericytes and astrocytes that can help to maintain the BBB integrity, especially in its recovery after insults [3,36,67,68,69,70].

Many in vivo and in vitro models [9,44,45,55,60,71,72,73] have been employed to investigate tumor cell adhesion and transmigration across vascular barriers, the two critical steps in tumor hematogenous metastasis [74]. The 2D BBB generated on the Transwell filter is the most convenient model for investigating tumor cell adhesion and transmigration. However, this 2D BBB under static conditions does not replicate the real physiological conditions in the cerebral microvessels. The blood flow not only brings fresh nutrients and carries away the cell-generated waste, it also induces shear stresses, which are important in endothelial responses to various stimulations via mechano-sensors and transcription factors [16,17] and in circulating cell adhesion and transmigration [18,19,20]. The 3D microchannel BBB produced in the current study had a circular-shaped cross-section and a diameter of ~100 μm, which enabled us to mimic the post-capillary venules in which tumor cells prefer to adhere after they escape the smaller-sized capillaries [55]. Compared to adhesion to the 2D BBB under static conditions, MB231 adhesion to the 3D BBB under normal physiologic flow increased by about 50%. After the treatment with 1nM VEGF, the MB231 adhesion increased to 3.4 folds of that without treatment. However, the increase caused by VEGF was only by 1.4 folds for the 2D BBB. This observation regarding MB231 adhesion under flow is consistent with that reported in Shen et al. [60] for MB435 cell (another type of breast cancer cell) adhesion in the post-capillary venules of the rat mesentery. They found that the number of adherent MB435 cells under normal flow (1 mm/s) was 1.2–1.4 folds that under a reduced flow with and without VEGF treatments [60]. Flow-induced shear stresses can enhance tumor cell adhesion to endothelial cells by activating cell adhesion molecules, including integrins [75], and inducing the clustering of adhesion molecules on the cell surface [76]. Hajal et al. reported that laminar flow in the microcirculation plays an important role in tumor cell adhesion and extravasation, which could determine the local metastatic potential of tumor cells [11]. Using the 3D BBB under flow, we found that treatment with heparinase III applied to only the BBB increased MB231 adhesion by 2.3 folds and treatment with orosomucoid reduced MB231 adhesion to 63% compared to that with no treatments. These observations are also consistent with that reported by Cai et al. [45] that heparinase III treatment increased MB231 adhesion by ~2.8 folds and orosomucoid treatment reduced the adhesion to ~54% in the post-capillary venules in the rat mesentery compared to that with no treatments.

The glycocalyx of tumor cells also mediates their adhesion and extravasation during metastatic dissemination [44,72]. As mentioned in earlier sections, heparinase III, VEGF, and orosomucoid were employed to modulate the production of glycocalyx, specifically, heparan sulfate (HS), in the BBB and MB231 cells. As was found in the recent study by Li et al. [44], although heparinase III reduces the HS production of both MB231 cells and the BBB, VEGF and orosomucoid have differential effects on the HS production of MB231 cells and that of the BBB. While VEGF increases/decreases the HS production of MB231 cells/the BBB, orosomucoid decreases/increases the HS production of MB231 cells/the BBB. For the 2D BBB under static conditions, heparinase III decreased the amount of HS to 63%, while it decreased the amount of HS to 34% for the 3D BBB under flow. The lower amount of HS in the 3D BBB enabled more MB231 adhesion, 2.3 folds that with no treatment, while the MB231 adhesion was only increased by 1.3 folds for the 2D BBB. The plasma protein orosomucoid is essential for the maintenance of stable microvessel solute permeability by enhancing the charge and organization of the endothelial glycocalyx [77]. Orosomucoid increased the amount of HS of the 2D BBB and that of the 3D BBB by 3.4 folds and 1.4 folds, and it decreased the MB231 adhesion to 68% and 63%, respectively, compared to that with no treatment. VEGF (a tumor secretion) reduced the amount of HS to 40% in the 3D BBB and to 29% in the 2D BBB compared to that without treatment. However, VEGF increased MB231 adhesion by 3.4 folds for the 3D BBB under flow and by 2.3 folds for the 2D BBB under static conditions. The flow seemed to have a larger effect than the amount of HS on MB231 adhesion. The flow-induced forces may activate the cell adhesion molecules on both MB231 cells and the BBB to promote MB231 adhesion, but the enhanced HS production at the BBB may decrease the flow effect on the cell adhesion molecules of the endothelial cells. HS proteoglycans (HSPGs) on the endothelial cell surface interact with various adhesion molecules and extracellular matrix (ECM) proteins, including fibronectin, laminin, thrombospondin, and collagen [78]. The syndecan family of HSPGs, particularly syndecan-1 and syndecan-4, are involved in regulating cell adhesion and migration through interactions with integrins and the actin cytoskeleton [79]. On the other hand, the HS on MB231 cells may behave as a cell adhesion molecule. The reduced HS on MB231 cells caused by orosomucoid likely weakens the increased adhesive ability caused by the flow.

During the last decade, in addition to primary cells and cell lines, human iPSCs (induced pluripotent cells) have been widely used to derive endothelial cells, astrocytes, pericytes, and neurons, which have been used to produce in vivo-like 3D human BBBs [2,7,33,49,67,80,81,82,83]. These 3D human BBB models do provide a crucial tool in understanding the role of cerebral vasculature in health and in disease, but they are expensive, and for some, access to a clean room may be needed for manufacturing the sophisticated tissue culture scaffolds. In our 3D microchannel BBB, we utilized inexpensive materials, including PDMS, collagen I, and microneedles. It can be conveniently constructed in any wet lab without using a clean room. We also used a cell line of hCMECs in our 3D BBB models, which are stable, easy to grow, and can maintain normal BBB phenotypes and the properties of primary cells [84,85]. Compared to the commonly used Transwell filters for 2D BBB production, which are more than $5 each, the cost for the material used in one 3D microchannel BBB is estimated to be ~$2. In addition to inexpensive materials, our 3D microchannel BBB production system is convenient and enables the continuous perfusion of the fresh cell culture medium just like in real blood circulation. Due to the small diameter and physiological flow rate, the required volume of the cell culture medium for producing one microchannel BBB is 8–10 mL, comparable to what is needed for producing one 2D BBB on a Transwell filter.

To determine how much the collagen gel used in this study (~5 mg/mL) contributed to the microchannel BBB permeability, we measured the permeability of the microchannel without hCMECs. Its permeability to NaFl was ~2.3 × 10^−4^ cm/s, and that to Dex-70k was ~8.2 × 10^−6^ cm/s, which are ~110 folds and ~42 folds those of the microchannel BBB, respectively. The resistance from the collagen gel to both solutes was about 1–2% of that of the BBB formed with hCMECs. This small resistance from the collagen gel can be neglected in calculating the microvessel BBB’s permeability to the solutes (Figure S3 in Supplement S3).

Although the currently produced 3D microchannel BBB has a comparable shape, size, and barrier functions to cerebral microvessels, because of the limitation in the tight junction protein expression at the hCMEC junction and the limitation in the imaging of a circular tube of ~100 μm, we could not perform the quantification of the tight junction proteins under various treatments for this 3D microchannel BBB model. In the future, we could use primary or iPSC-derived human cerebral microvascular endothelial cells for quantifying the tight junction protein expression under various treatments. Due to the same imaging limitation in the current study, we could not reveal the details of the HS alteration and identify the upregulated cell adhesion molecules during MB231 adhesion under flow in the 3D microchannel BBB. Future studies will be conducted to investigate these molecular and cellular mechanisms under various conditions. In addition, this 3D BBB model does not have surrounding astrocytes and pericytes, which not only are components of the BBB but also play an important role in maintaining the BBB integrity and function as reported in [36]. The current design can be modified to seed the astrocytes and pericytes first on the luminal surface of the collagen gel before seeding hCMECs in a larger-diameter microchannel. In addition, the current 3D microchannel BBB model can only simulate a single microvessel with a ~100 μm diameter; the complex vascular networks observed in brain tissue are difficult to create using the current platform. More sophisticated platforms will be developed to mimic the real cerebral microvasculature in the future.

## 5. Conclusions

We have developed a 3D human microchannel BBB model in a PDMS–collagen hydrogel device under physiological flow. This microchannel BBB has a circular-shaped cross-section with a diameter of ~100 μm. It has the same permeability to both small and large solutes as that of rat cerebral microvessels and can replicate the response to a plasma protein, orosomucoid, a cytokine, VEGF, and an enzyme, heparinase III, in either rat cerebral or mesenteric microvessesels in terms of permeability and glycocalyx (heparan sulfate) production. It can also replicate the tumor cell (MB231) adhesion in rat mesenteric microvessels under no treatment and under treatments with VEGF, orosomucoid, and heparinase III. Because of difficulties in accessing human cerebral microvessels, this cost-effective and easy to assemble 3D human BBB model can be applied to investigate BBB-modulating mechanisms in health and in disease as well as to develop therapeutic interventions targeting tumor metastasis to the central nervous system.

## Data Availability

The data are contained within the article.

## Supplementary Materials

The following supporting information can be downloaded at: https://www.mdpi.com/article/10.3390/cells14060456/s1, Supplement S1: Step-by-step instructions for the production of a 3D PDMS–hydrogel microchannel and a 3D microchannel BBB model; Supplement S2: Detailed instructions for the determination of the solute permeability of the microchannel BBB using a fluorescence microscope; Supplement S3: Spreading images of fluorescent solutions in the empty microchannel and in the microchannel BBB.

## Appendix A

Figure A1 shows the calibration for the concentration vs. intensity curves for sodium fluorescein and FITC-Dex-70k using the same imaging system as for the solute permeability measurement. Figure A2 is from the study by Li et al. (2024) [44], showing confocal images of the modulation of HS in a 2D BBB and MB231 cells.
