# Supplementary material for: A Cost-Effective and Easy to Assemble 3D Human Microchannel Blood–Brain Barrier Model and Its Application in Tumor Cell Adhesion Under Flow
Source: Cells. 2025 Mar 19;14(6):456. doi: 10.3390/cells14060456 (PMC11941619; doi:10.3390/cells14060456)
Supplement: Supplementary file 1 [file cells-14-00456-s001.zip › cells-3428955-supplementary.pdf]

Supplement S1

The production of a 3D PDM–hydrogel microchannel and a 3D microchannel BBB model

A. The Production of a 3D PDMS–Hydrogel Microchannel

1. Preparation of PDMS

A polydimethylsiloxane (PDMS; Sylgard, Dow Corning) pre-polymer was mixed with a curing agent in a 10:1 (w/w) ratio by stirring for at least 3 minutes. The amounts of the PDMS and curing agent were measured using a balance. The PDMS mixture was degassed in a vacuum chamber for at least 30 minutes to remove the air bubbles induced during mixing.

2. Glass Coverslip Cleaning

A glass coverslip (22 x 22 mm, Dow Corning) was thoroughly cleaned with acetone or ethanol (70%), followed by deionized water. It was then dried using high-speed airflow.

3. Microneedle Coating

A 1% BSA/PBS solution was used to coat a 120 µm acupuncture needle (SEIRIN, Thermo Fisher Scientific, Waltham, MA) overnight at 4°C.

4. PDMS Base Formation

In a hexagonal container, the liquid degassed PDMS mixture was poured onto the cleaned coverslip with the 10 mm microneedle segment placed on its surface. The thickness of the PDMS mixture in the container was ~3 mm. Then, the PDMS was cured for 35 minutes at 100°C in an oven or overnight at 37°C in an incubator. Once solidified, the PDMS was carefully cut along the edge of the coverslip to create a PDMS base with the coverslip at the bottom. The PDMS base formed with the microneedle inside is shown in **Fig. S1** (left).

5. Microneedle Removal

The microneedle was gently pulled out, creating a microchannel in the PDMS device.

6. Drilling the Inlet

An 18G tubing adapter was used to drill a hole perpendicularly into the PDMS base, forming an inlet. The hole depth needed to be carefully controlled to avoid breaking the coverslip but enable the inlet to connect with the microchannel.

7. Collagen Hydrogel Preparation

Collagen type I from a rat tail (Advanced Biomatrix, Thermo Fisher Scientific, Waltham, MA) was neutralized and diluted to a concentration of approximately 5 mg/ml. A total of 1 mM genipin (Sigma-Aldrich, St. Louis, MO, USA) was added for crosslinking to stabilize the collagen hydrogel.

8. Formation of Collagen Gel-Filled Segment in PDMS Base

The collagen solution was mixed by pipetting it up and down five times to ensure uniformity. Then, the collagen mixture was added into the PDMS microchannel with a pre-cut segment of ~3mm x 3mm, while the microneedle was still in place.

9. Collagen Gelation

The PDMS device with a segment filled with collagen gel was incubated overnight at 37°C. After incubation, the needle was slowly pulled out to create a microchannel in the collagen gel.

10. Device Placement

The PDMS–hydrogel microchannel device was placed into a Petri dish with a 10 cm diameter. **Figure S1** shows the perfusion of a fluorescent solution into microchannels without (middle) and with (right) a collagen gel segment via PE-50 tubing inserted into the inlet of the microchannel.

B. The Generation of a 3D microchannel BBB

1. Inlet Connection

The inlet was connected to PE-50 tubing (BD, Thermo Fisher Scientific, Waltham, MA) for perfusion, which was driven by a syringe pump (NE-1800, New Era Pump Systems, NY, USA). All tubing and syringes were sterilized.

2. PBS and Medium Perfusion

The microchannel was first perfused with PBS for at least 1 hour to remove residual genipin and was perfused overnight with a cell culture medium.

3. Preparation of EGMTM-2 MV with Dextran

To prepare EGMTM-2 MV with 8% dextran-70k, dextran-70k was added to the medium and thoroughly dissolved. The solution was then sterilized by filtration.

4. Cell Suspension Preparation

A suspension of human cerebral microvascular endothelial cells (hCMECs) at a density of 4-5 million cells/ml was prepared in the EGMTM-2 MV culture medium containing 8% dextran-70k.

5. Cell Seeding

The cell suspension was slowly perfused into the microchannel via the inlet using a pipettor with a 10 µL pipette.

6. Cell Attachment

After checking that the cell suspension filled up the microchannel using a microscope, the device was incubated and flipped every 15 minutes to ensure even seeding across the microchannel surface.

7. Medium Change

After 1 hour of incubation, the cell suspension was replaced with a fresh EGMTM-2 MV medium to remove excess cells.

8. The Formation of a 3D Microchannel BBB

Six hours after cell seeding, the microchannel was connected to the PE-50 tubing system for cell culture medium perfusion at a rate of 1.0-1.3 µl/min using the syringe pump. Care was taken to ensure there were no bubbles in the system. Cells were monitored and waste from the outlet

was removed daily. A 3D microchannel BBB was formed in 4-5 days under a continuous physiological flow at 1.0-1.3 µl/min.

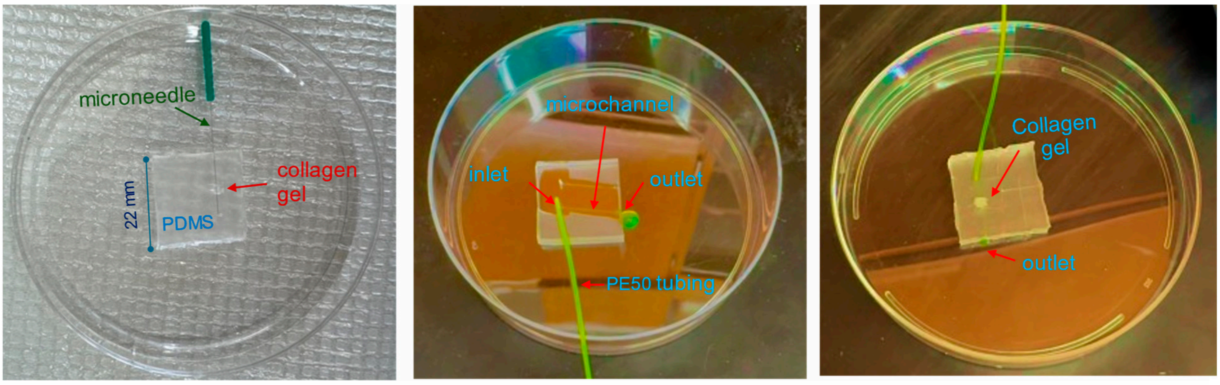

Fig. S1. A microneedle inside a PDMS base with a ~ 3mm x 3mm x 3mm segment filled with collagen gel to form a microchannel after being pulled out (left). A fluorescent solution is perfused via PE50 tubing into microchannels without (middle) and with a collagen gel-filled segment (right).

Supplement S2

The determination of the solute permeability of the microchannel BBB using a fluorescence microscope

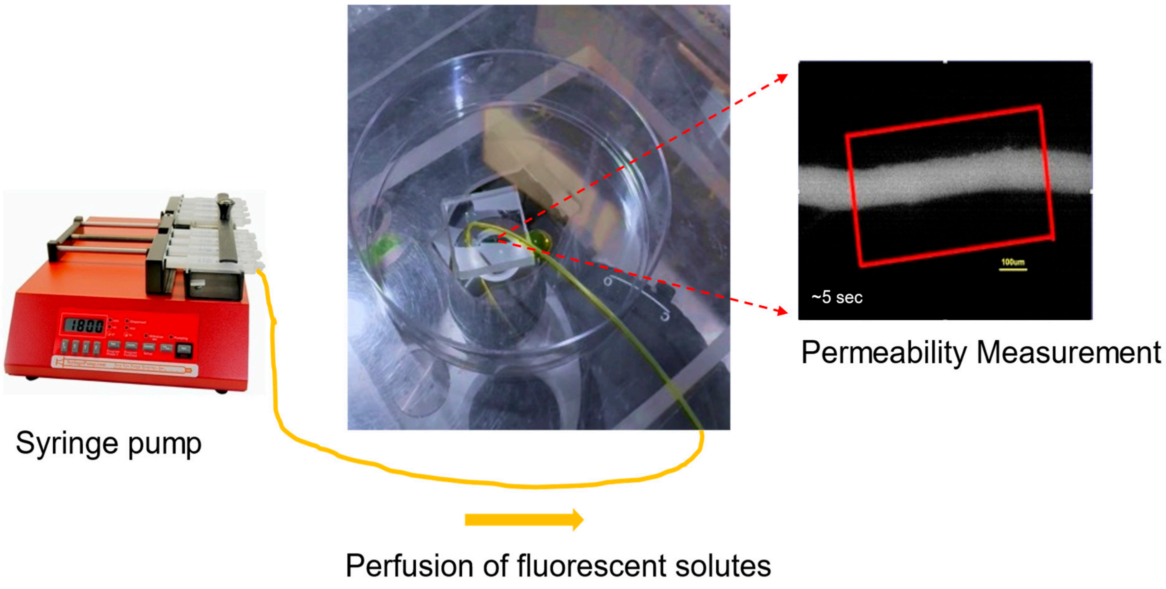

Fig. S2A. Illustrations showing the determination of the solute permeability of a microchannel BBB. The microchannel BBB was viewed using an inverted Nikon-TE2000S imaging system with a 20x/NA0.75 objective lens. When the solution of the fluorescently labeled solutes in 1% BSA–Ringer solution was perfused into the microchannel BBB using a syringe pump at a rate of ~1.5μl/min, simultaneously, a highly sensitive 14-bit digital camera was used to collect images at a rate of 2-4 frames/sec for 5-60 sec for each size of the solutes in our study. The excitation/emission wavelengths were 490 nm/520 nm for sodium fluorescein (NaFl) and FITC-Dex-70k. The image collection time needed to be kept as short as possible to avoid damage to the BBB induced by the fluorescent light. The image on the right was taken ~5 sec after the NaFl solution filled up the lumen of the microchannel BBB.

Fig. S2B shows how to use the collected fluorescence images to determine the diffusive solute permeability of the microchannel BBB. In our experiments, the perfusion rate was low (~1.5μl/min), and the outlet was exposed to the atmosphere. The hydrostatic pressure in the microchannel was estimated to be less than 2 cmH_2O, which contributed to negligible convective transport for trans-channel wall solute transport. The transmural solute transport was almost a pure diffusion process. The diffusive solute permeability of a permeable microchannel (like the microchannel BBB or a real microvessel) is defined as the solute outflow rate per unit channel surface area divided by the driving force (the concentration difference across the channel wall). For a circular-shaped microchannel with a radius of r, if P represents the diffusive solute permeability, the solute concentration is C, the concentration inside the lumen of the channel is denoted by C_L, and that outside the wall in the tissue (or gel) is C_t. The flux across the channel wall is $\frac{d(C\pi r^2 L)}{dt} \times \frac{1}{2\pi r L} = \frac{dC}{dt} \times \frac{r}{2}$. L here is the length of the microchannel. Therefore, $P = \frac{\frac{dC}{dt}}{\Delta C} \times \frac{r}{2}$.

Here, $\Delta C = C_L - C_t$ is the concentration difference across the microchannel wall, which is the driving force for the solute diffusion across the wall. **Figure A1** in the Appendix shows that the concentration of our fluorescent solutions was proportional to the intensity measured using our imaging system. $C = A \times I$. Here, I is the fluorescence intensity, and A is the proportional coefficient, which is the constant for a linear relation between the concentration and intensity of the fluorescent solution. $\Delta C = A \times \Delta I$. $\Delta I = I_L - I_t$. I_L and I_t are the fluorescence intensity in the lumen and that outside the wall in the tissue (gel), respectively. At the beginning of the solute perfusion, the driving force is $\Delta I_0 = I_{L0} - I_{t0}$, which is the step increase in the intensity when the solute just fills up the channel lumen, as shown in **Fig. 4B**. The solute permeability of the microchannel can be determined as $P = \frac{1}{\Delta I_0} \times (\frac{dI}{dt})_0 \times \frac{r}{2}$. Here, $(\frac{dI}{dt})_0$ is the initial rate of the increase in the fluorescence intensity after the solute fills the lumen and begins to accumulate in the gel (Huxley et al, 1987; Yuan et al, 2009; Cai et al, 2012; Shi et al, 2014).

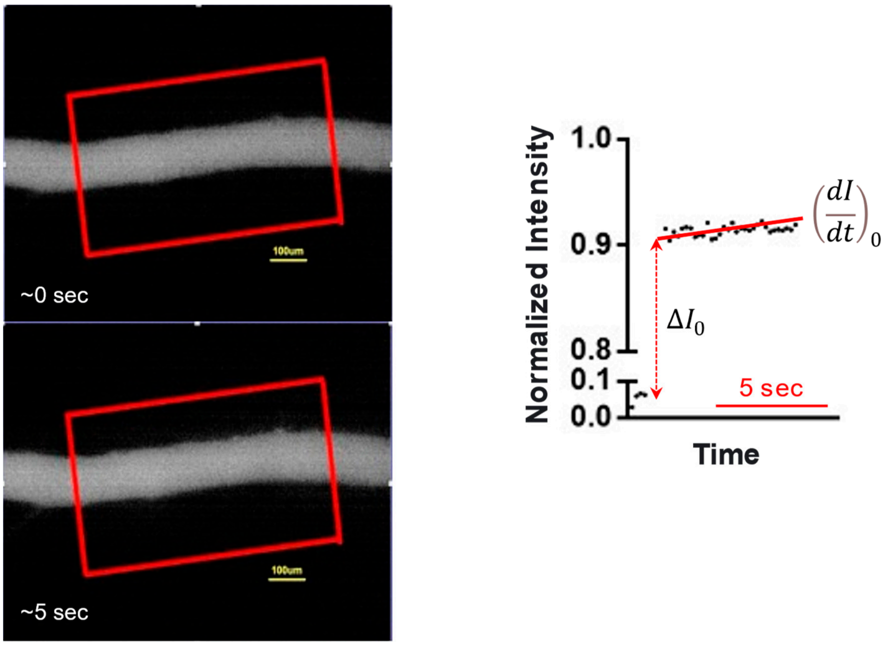

Fig. S2B. Two images of the fluorescent solutes (NaFl or dye) in the microchannel BBB and its surrounding gel (left). One was taken right after the channel lumen was filled with the dye (~0 sec) and another at ~5 sec after the lumen was filled with the dye. The total fluorescent intensity (I) in the measuring window (the region enclosed by red lines, ~300-500 μm long and ~200-350 μm wide) was calculated by using NIH ImageJ. The size of the measuring window was chosen to satisfy the condition that the dye does not spread out of the window during the time taken for the P measurement while collecting as long a segment as possible in that channel. The curve of the intensity vs. perfusion time (I vs. t) was plotted (right). The intensity was normalized by the maximum intensity in the course of the measurement. The slope of the initial intensity increase curve $(dI/dt)_0$ and the step increase in the intensity when the solute just fills up the channel lumen (ΔI_0), as well as the radius of the channel (r), were used to calculate the solute permeability of the microchannel BBB: $P = \frac{1}{\Delta I_0} \times (\frac{dI}{dt})_0 \times \frac{r}{2}$. For this microchannel BBB, the permeability to NaFl was determined as ~1.9 x 10^{-6} cm/s.

To use the above equation to calculate the solute permeability of a microchannel, in addition to a linear relation between the solute concentration and the intensity, there is another requirement that the imaging system used can detect all the fluorescence in the volume of interest. This means that the depth of light collection of the imaging system should be large enough. We determined the depth of light collection of our imaging system with a 20x/NA0.75 lens following the method described in studies by Huxley et al., 1987, Yuan et al., 2009, and Shi et al., 2014. Briefly, six chambers with depths of 20, 40, 80, 100, 170, and 340 μm were constructed to measure the fluorescence intensity of the NaFl solution under the same experimental settings as those for the permeability measurements. The chambers with a 20-100 μm depth were constructed by sandwiching membranes with a 10 μm thickness from 24 Transwell filters (Corning, Corning, NY) between two coverslips. The 170 and 340 μm deep chambers were formed by placing one or two

small 22 x 22 mm coverslips with a 170 μm thickness between two large 22 x 50 μm coverslips. The concentration–depth product was kept as a constant to make the fluorescein solution for the chambers of various depths. The fluorescence intensity was measured for a window area of 436 μm × 334 μm by focusing on the top surface of the chamber. The intensity was almost the same for the chambers with a depth from 20 to 100 μm but decreased as the chamber depth increased from 170 to 340 μm (**Fig.S2C**). The curve fitting for the measured intensity in the chambers of different depths was performed by using a light collection index function (Yuan et al., 1993). The depth of light collection in our system was ~105 μm (z_0 = 105 μm in the best-fitting index function), i.e., within this depth range, our imaging system could collect all the light. Beyond this depth, our system could collect only partial or no light. Within this depth range of light collection, the fluorescence intensity was proportional to the total number of fluorescent molecules and was independent of the chamber depth. Using our current imaging setting, we could only correctly determine the solute permeability of a microchannel with a diameter of ~100 μm or less. For larger-sized microchannels, an imaging system with a lower magnification and/or lower numerical aperture lens should be used.

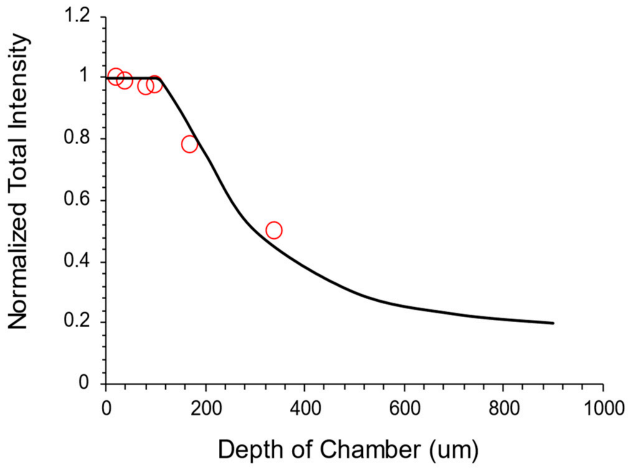

Fig. S2C. Determination of the depth of light collection. Samples of a sodium fluorescein solution were prepared such that the concentration–depth product was maintained as a constant. The total fluorescence intensity, which was normalized by the value for the 20 μm depth chamber, was plotted for the chambers with depths of 40, 80, 100, 170, and 340 μm. The smooth line is the curve fitting the total intensity determined using a light collection index function from Yuan et al. (1993).

Supplement S3

Figure S3 demonstrates the spreading images of the fluorescent solute (dye) in the microchannel without hCMECs (microchannel) and those in the microchannel with hCMECs (microchannel BBB). We can see that within ~5 sec after perfusion, there is plenty of NaFl (MW = 376, Stokes radius of ~0.45 nm) spreading out of the empty microchannel but not much out of the microchannel BBB, which is hard to detect with the eyes from the image, but it could be detected by the imaging analyzing software. The reason was that the permeability of the empty microchannel to NaFl was in the order of 10^{-4} cm/s and that of the microchannel BBB was in the order of 10^{-6} cm/s. Similarly, for the large molecule Dex-70k (Stokes radius of ~3.5 nm), after ~30 sec of perfusion, there was significant spreading surrounding the empty microchannel, while there was not spreading visible to the eyes in the image for the microchannel BBB, but it could be detected by the imaging analyzing software. The cause was that the permeability of the empty microchannel to Dex-70k was in the order of high 10^{-6} cm/s and that of the microchannel BBB was in the order of 10^{-7} cm/s.

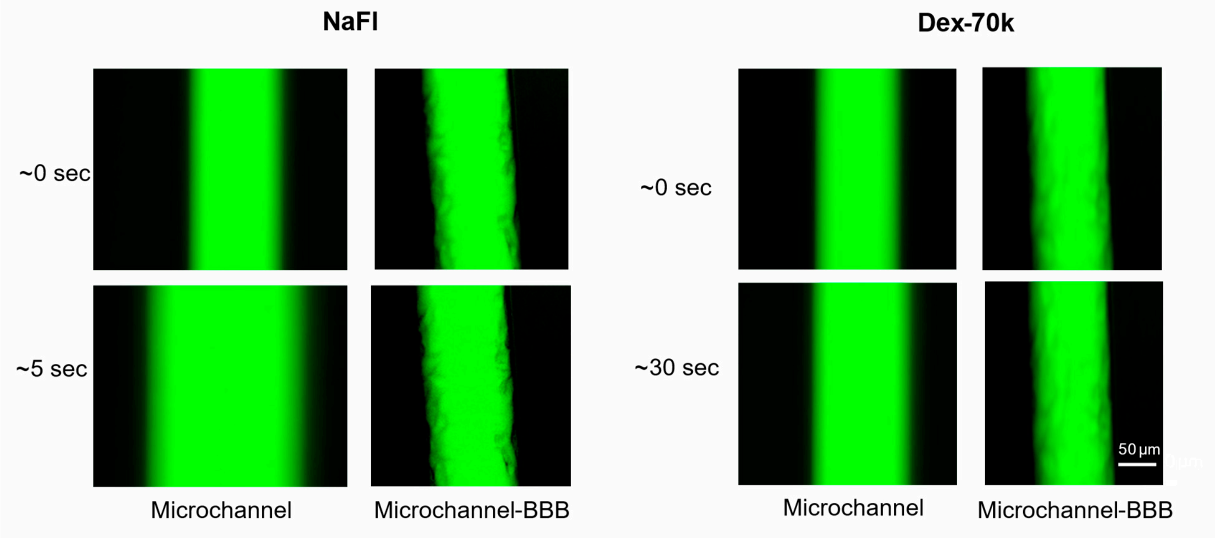

Fig. S3. Spreading images of NaFl (left) and Dex-70k (right) in a microchannel without hCMECs (microchannel) and in a microchannel with hCMECs (microchannel BBB). ~0 sec indicates the moment when the dye just fills up the microchannel lumen.
